# Care patterns of patients with chronic fibrosing interstitial lung disease (ILD) with a progressive phenotype

**DOI:** 10.1186/s12890-022-01953-9

**Published:** 2022-04-23

**Authors:** Mona Nili, David Singer, Maya Hanna

**Affiliations:** Boehringer Ingelheim Pharmaceuticals, Inc., 900 Ridgebury Rd, Ridgefield, CT 06877 USA

**Keywords:** Chronic fibrosing ILD with a progressive phenotype, Care pattern, Medication use

## Abstract

**Background:**

Interstitial lung diseases (ILDs) include a variety of parenchymal lung diseases. The most common types of ILDs are idiopathic pulmonary fibrosis (IPF), autoimmune ILDs and hypersensitivity pneumonitis (HP). There is limited real world data on care patterns of patients with chronic fibrosing ILDs with a progressive phenotype other than IPF. Therefore, the aim of this study is to describe care patterns in these patients.

**Methods:**

This retrospective cohort study used claims data from 2015 to 2019 from the Optum Research Database. The study population included adults (≥ 18 years old) with at least two diagnosis codes for fibrosing ILD during the identification period (1OCT2016 to 31DEC2018). A claim-based algorithm for disease progression was used to identify patients likely to have a progressive fibrotic phenotype using progression proxies during the identification period. Index date was the first day of progression proxy identification after fibrosing ILD diagnosis. Patients were required to have continuous enrollment for 12 months before (baseline) and after (follow-up) index date. Patients with an IPF diagnosis were excluded. Descriptive statistics were used to describe the patient population and care patterns.

**Results:**

11,204 patients were included in the study. Mean age of the patient population was 72.7 years, and 54.5% were female. Unclassified ILDs (48.0%), HP (25.2%) and autoimmune ILDs (16.0%) were the most common ILD types. Other respiratory conditions were prevalent among patients including chronic obstructive pulmonary disease (COPD) (58.9%), obstructive sleep apnea (OSA) (25.0%) and pulmonary hypertension (9.8%). During baseline, 65.3% of all patients had at least one pulmonology visit, this proportion was higher during follow-up, at 70.6%. Baseline and follow-up use for HRCT were 39.9% and 48.8%, and for pulmonary function tests were 43.7% and 48.5% respectively. Use of adrenal corticosteroids was higher during follow-up than during baseline (62.5% vs. 58.0%). Anti-inflammatory and immunosuppressive medication classes were filled by a higher percentage of patients during follow-up than during baseline.

**Conclusions:**

Comprehensive testing is essential for diagnosis of a progressive phenotype condition, but diagnostic tests were underutilized. Patients with this condition frequently were prescribed anti-inflammatory and immunosuppressive medications.

**Supplementary Information:**

The online version contains supplementary material available at 10.1186/s12890-022-01953-9.

## Background

Interstitial lung disease (ILD) is a broad term that describes over 200 diverse lung disorders, including idiopathic pulmonary fibrosis (IPF), autoimmune ILD, hypersensitivity pneumonitis (HP) and sarcoidosis [1]. Typical symptoms of ILD are cough and dyspnoea, as well as decreased lung capacity [2]. IPF is the most common idiopathic ILD and represents the prototype of progressive fibrosing ILD depicted by lung function decline and early mortality [3]. In addition to IPF, 13–40% of patients with other fibrosing ILDs can develop progressive pulmonary fibrosis during their disease course [4]. Chronic fibrosing ILD with a progressive phenotype, despite manifesting in patients with a variety of underlying conditions, is driven by overlapping pathogenic mechanisms including lung parenchymal injury, TGF-mediated fibroblast activation, and myofibroblast accumulation [5].

There is a lack of consensus among physicians on how to diagnosis and treat patients with chronic fibrosing ILD with a progressive phenotype [6]. Diagnosis and treatment of patients with a progressive phenotype is complex, often requiring a multi-disciplinary team of physicians, including pulmonologists and rheumatologists [7]. High-resolution computed tomography (HRCT) is the main diagnostic tool for differential diagnosis. Pulmonary function testing (e.g. forced vital capacity (FVC), diffusing capacity of the lungs for carbon monoxide [DLco]) and tissue biopsy are also used for diagnosis [8].

After diagnosis of a progressive phenotype, patients are usually treated on an empirical basis by using corticosteroids as first-line treatment, often in conjunction with other immunosuppressive medications [9]. Using empirical treatment is a common practice in many developed countries, even though this treatment approach does not have enough clinical evidence in terms of efficacy [10]. In addition, some immunosuppressive medications (e.g. antitumor necrosis factor (TNF) α) may be associated with deteriorating ILD effects [11]. In March 2020, Food Drug Administration approved the first therapeutic agent, nintedanib, for treatment of patients with chronic fibrosing ILDs 12]. Approval of nintedanib could significantly improve the treatment pattern of patients with a progressive phenotype.

With limited evidence on care patterns of patients with chronic fibrosing ILD with a progressive phenotype, there is a need to better understand care and treatment of this condition. This study will focus on non-IPF patients as the care pattern of IPF was extensively investigated in previous studies. To better understand the real-world treatment patterns in chronic fibrosing ILD with a progressive phenotype, this study used administrative claims data to investigate the current care patterns in patients with chronic fibrosing ILD with a progressive phenotype.

## Methods

### Study design

This study was an observational retrospective cohort study using existing administrative claims data.

### Data source

This study used administrative claims data from 2015 to 2019 from the Optum Research Database (ORD). The ORD is geographically diverse across the US and contains deidentified medical and pharmacy claims data and linked enrollment information for individuals enrolled commercial and Medicare Advantage health plans. Medical claims in the ORD include, but are not limited to, dates and place of service (e.g., inpatient, outpatient, and emergency department visits), diagnoses, procedures, and detailed information on hospitalizations including admission and discharge dates. Pharmacy claims in the ORD include complete outpatient prescription drug information, which includes the costs of mail-order drugs, injectables, drugs from specialty pharmacies, and all standardized prescription-level fields collected on a typical pharmacy claim (e.g., date of fill or refill, drug name and class, strength, quantity, and days supply).

### Study population

**Fig. 1:**
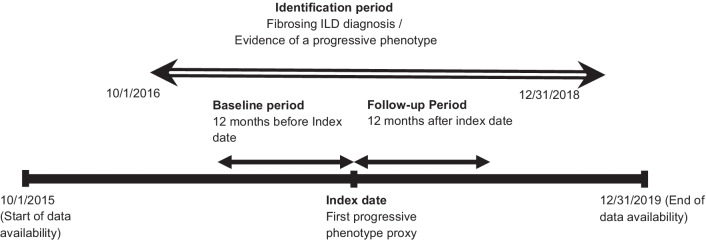
Study design

The study included commercial and Medicare Advantage with Part D (MAPD) health plan enrollees diagnosed with chronic fibrosing ILD and a progressive phenotype proxy from 01 to 2016 through 31 December 2018 (Fig. 1). The index date of this study was the date of a claim for a proxy for the progressive phenotype. Chronic fibrosing ILD was identified for the individuals with at least two medical claims on separate dates, within 30–365 days, with diagnosis codes for lung fibrosis (ICD-10-CM codes: J8410, J8489, J84111, J84113, J8409, J849, and M3481), identification period [13, 14]. As there was not any diagnostic or procedure codes specific to a progressive phenotype during study identification period, this study used previously validated proxies for progressive phenotype identification [13, 14]. In order to identity individuals with a progressive phenotype, the following proxies were used: (1) At least two pulmonary function tests within 90 days of each other; (2) At least two high-resolution computed tomography (HRCT) scans within 360 days of each other; (3) At least three chest computed tomography scans within 360 days of each other; (4) At least two oxygen titration tests within 90 days of each other; (5) At least one claim for oxygen therapy; (6) At least one respiratory hospitalization; (7) At least one claim for palliative care; (8) At least one claim for a lung transplant; (9) At least one new claim for an immunosuppressive medication; (10) At least one claim for oral corticosteroids with a dose greater than 20 mg per day (Additional file 1: Appendix) [13, 14]. The date of first claim was used as the index date for those proxies that required more than one claim for identification.

This study excluded those who were less than 18 years of age at index date. Other exclusions included (1) less than 12 months of continuous enrollment in medical and pharmacy benefits before and after the index date (enrollment gap less than 30 days was allowed); (2) at least one IPF diagnosis claim (ICD-10-CM: J84.112) during study period; (3) more than one insurance type during study period.

### Measures

Demographic and clinical characteristics of patients were measured at the index date or during the 12-month baseline period, and included age, gender, insurance type, geographic region, and race/ethnicity. Baseline clinical characteristics included Charlson comorbidity score, ILD type, and comorbidities of interest, identified by using International Classification of Diseases, 10th Revision, Clinical Modifications (ICD-10-CM) codes (Additional file 1: Appendix).

Baseline and follow-up patterns of care (i.e. diagnostic tools, specialist visits, and prescribed medications use) were assessed for all patients. As patients with autoimmune ILD (rheumatoid arthritis, systemic lupus erythematosus, dermatomyositis, polymyositis, systemic sclerosis, and mixed connective tissue disease) may have different types of specialist visits and medications, this study reported these measures separately for this group of patients. Use of HRCT and pulmonary function test were captured by using Current Procedural Terminology, 4th Edition (CPT-4) codes (Additional file 1: Appendix). Lung biopsy was identified by using ICD-10 Procedure Coding System (ICD-10-PCS) (Additional file 1: Appendix). Moreover, pulmonologists and rheumatologist visits were assessed based on the provider specialty category. Use of selected medications were identified by using National Drug Code (NDC) (Additional file 1: Appendix), reported as the percentage of patients with at least one prescription. The most common classes of medication were identified based on Multum level 2 categories. Select classes of medications were (1) oral corticosteroids (OCS) (i.e., prednisone, methylprednisolone, hydrocortisone prednisolone, dexamethasone, cortisone acetate, betamethasone), (2) Biologic disease-modifying antirheumatic drugs (DMARDs) (i.e., rituximab, tocilizumab, abatacept, denosumab, etanercept, adalimumab, infliximab), (3) Non-biological DMARDs (i.e., methotrexate, hydroxychloroquine, leflunomide, sulfasalazine, azathioprine, chloroquine phosphate, penicillamine) and (4) Immunomodulators (i.e., cyclosporine, tacrolimus, mycophenolate mofetil, sirolimus, cyclophosphamide, everolimus).

### Statistical analysis

All study variables were analyzed descriptively. Numbers and percentages were provided for categorical variables; means and standard deviations were provided for continuous variables. All analyses were performed using Instant Health Data (IHD) software (Panalgo, Boston MA, USA) and R, version 3.2.1 (R Foundation for Statistical Computing, Vienna, Austria).

## Results

### Study sample

**Fig. 2:**
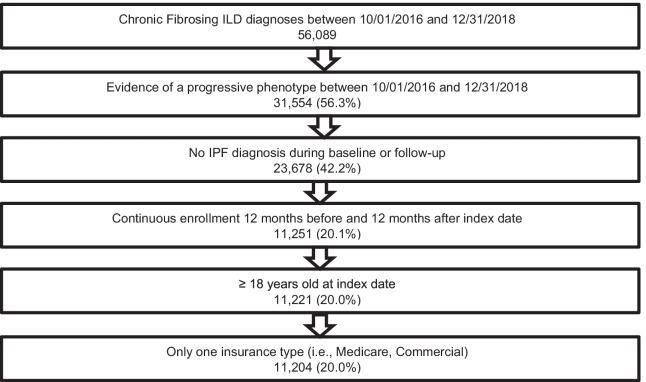
Patient attrition, Optum Research Database during 2015–2019

A total of 11,204 patients with chronic fibrosing ILD with a progressive phenotype met eligibility criteria and were included in the study (Fig. 2). The mean age of the patient population was 72.7 years and 54.5% were female. The majority of the population was Caucasian (68.5%) and had Medicare coverage (84.3%) (Table 1). Unclassified ILDs (48.0%), hypersensitivity pneumonitis (25.2%) and autoimmune ILDs (16.0%) were the most common ILD types. Some common comorbid conditions among this population were hypertension (81.6%), diabetes (37.5%), and heart failure (30.3%). Other respiratory conditions were also prevalent including chronic obstructive pulmonary disease (COPD) (58.9%), obstructive sleep apnea (OSA) (25.0%), and pulmonary hypertension (9.8%) (Table 1).

**Table 1:** Patient characteristics

	All patients (N = 11,204)
Age, mean (± SD)	72.7 (± 12.1)
Gender, female, n (%)	6110 (54.5)
Race/ethnicity, n (%)	
Caucasian	7672 (68.5)
African American	1390 (12.4)
Asian	227 (2.0)
Hispanic	892 (8.0)
Unknown	1023 (9.1)
Geographic region, n (%)	
Midwest	2729 (24.4)
Northeast	1498 (13.4)
South	4936 (44.1)
West	2035 (18.2)
Unknown	6 (< 0.1)
Insurance type, n (%)	
Commercial	1758 (15.7)
Medicare	9446 (84.3)
Charlson Comorbidity Score, mean (± SD)	3.3 (± 2.4)
Comorbid conditions, n (%)	
Hypertension	9146 (81.6)
COPD	6610 (58.9)
Diabetes	4202 (37.5)
Heart failure	3400 (30.3)
Chronic renal diseases	3237 (28.9)
Obstructive sleep apnea	2854 (25.5)
Pulmonary hypertension	1102 (9.8)
History of tobacco use	5790 (51.7)
ILD type, n (%)	
Unclassified ILD^a^	5381 (48.0)
Hypersensitivity pneumonitis	2825 (25.2)
Autoimmune ILD	1789 (16.0)
Sarcoidosis	372 (3.3)
Idiopathic nonspecific interstitial pneumonia	73 (0.6)
Autoimmune ILD and hypersensitivity pneumonitis	640 (5.7)
Autoimmune ILD and sarcoidosis	36 (0.3)
Hypersensitivity pneumonitis and sarcoidosis	88 (0.8)

*COPD* chronic obstructive pulmonary disease, *ILD* interstitial lung disease, *HRCT* high-resolution computed tomography, *SD* standard deviation

^a^Unclassified ILD included ILD types other than hypersensitivity pneumonitis, autoimmune ILD, sarcoidosis or idiopathic nonspecific interstitial pneumonia

### Patterns of care

During baseline, 65.3% of all patients had at least one pulmonology visit, this proportion was higher during follow-up, at 70.6% (Table 2). 42% of patients with an autoimmune condition had at least one pulmonology and one rheumatology visit during baseline, and this proportion was 48.1% during follow-up. Baseline use of diagnostic tests was generally lower than follow-up except for lung biopsy [HRCT (39.9% and 48.8%), pulmonary function tests (43.7% and 48.5%), and lung biopsy (12.7% vs. 9.8%)] (Table 3). The baseline use of the most common class of medications, adrenal cortical steroids, was higher during follow-up for the overall population (58.0% vs. 62.5%) and for those with autoimmune conditions (70.4% vs. 74.7%) (Table 4). Among all patients, the oral corticosteroids class (baseline: 45.8% and follow-up: 60.2%) was the most used anti-inflammatory and immunosuppressive medication class. Use of oral corticosteroids was higher among patients with autoimmune conditions (baseline: 58.7% and follow-up: 68.5%). Anti-inflammatory and immunosuppressive medication classes were filled by a higher percentage of the population during follow-up than during baseline (Table 4). During baseline, 6.9% of patients with an autoimmune condition had at least one prescription of mycophenolate mofetil; this number almost doubled (13.0%) during follow-up. Moreover, the percentage of use of azathioprine, cyclophosphamide, IVIG, rituximab, and tacrolimus increased during follow-up in comparison with the percentage of use during baseline. In contrast, patients with an autoimmune condition used methotrexate and anti-tumor necrosis factor-α with a lower percentage during follow-up compared to baseline (Table 4).

**Table 2:** Patterns of specialty visit

	All patients (N = 11,204)		Autoimmune conditions (N = 2,465)	
	Baseline n (%)	Follow-up n (%)	Baseline n (%)	Follow-up n (%)
At least one pulmonologist visit	7315 (65.3)	7911 (70.6)	1730 (70.2)	1923 (78.0)
At least one pulmonologist visit and zero rheumatologist visits	–	–	700 (28.4)	736 (29.9)
At least one rheumatologist visit and zero pulmonologist visits	–	–	340 (13.8)	234 (9.5)
At least one pulmonologist visit and at least one rheumatologist visit	–	–	1030 (41.8)	1187 (48.1)
Zero pulmonologist visits and zero rheumatologist visits	–	–	396 (16.0)	309 (12.5)

**Table 3:** Patterns of diagnostic tool use

	Baseline n (%)	Follow-up n (%)
High resolution computed tomography	4473 (39.9)	5468 (48.8)
Pulmonary function test	4901 (43.7)	5430 (48.5)
Lung biopsy	1423 (12.7)	1100 (9.8)

**Table 4:** Patterns of top and selected classes and medications use

	All patients (N = 11,204)		Autoimmune conditions (N = 2,465)	
	Baseline n (%)	Follow-up n (%)	Baseline n (%)	Follow-up n (%)
Percentage of patients with at least one prescription for most common classes of medications				
Adrenal cortical steroids	6502 (58.0)	6999 (62.5)	1737 (70.4)	1842 (74.7)
Analgesics	6083 (54.3)	5847 (52.2)	1550 (62.8)	1494 (60.6)
Bronchodilators	5857 (52.3)	6104 (54.5)	1262 (51.2)	1299 (52.7)
Antihyperlipidemic agents	5620 (50.2)	5644 (50.4)	1053 (42.7)	1085 (44.0)
Beta-adrenergic blocking agents	4446 (39.7)	4671 (41.7)	809 (32.8)	889 (36.0)
Proton pump inhibitors	4438 (39.6)	4831 (43.1)	1143 (46.3)	1223 (49.6)
Percentage of patients with at least one prescription for selected classes of medications				
Oral corticosteroids	5135 (45.8)	6,750 (60.2)	1,448 (58.7)	1,690 (68.5)
Non-biological DMARDs	2596 (23.2)	2,973 (26.5)	1,070 (43.4)	1,130 (45.8)
Immunomodulators	485 (4.3)	891 (7.9)	320 (13.0)	568 (23.0)
Biological DMARDs	455 (4.1)	551 (4.9)	296 (12.0)	335 (13.6)
Percentage of patients with at least one prescription for selected medications				
Adalimumab	59 (0.5)	53 (0.5)	52 (2.1)	46 (1.9)
Azathioprine	218 (1.9)	372 (3.3)	151 (6.1)	246 (10.0)
Cyclophosphamide	20 (0.2)	31 (0.3)	9 (0.4)	14 (0.6)
Etanercept	46 (0.4)	41 (0.4)	42 (1.7)	38 (1.5)
Intravenous immunoglobulin	80 (0.7)	126 (1.1)	34 (1.4)	45 (1.8)
Infliximab	27 (0.2)	18 (0.2)	25 (1.0)	16 (0.6)
Methotrexate	372 (3.3)	309 (2.8)	314 (12.7)	238 (9.6)
Mycophenolate mofetil	244 (2.2)	470 (4.2)	170 (6.9)	320 (13.0)
Rituximab	35 (0.3)	107 (1.0)	22 (0.9)	67 (2.7)
Tacrolimus	84 (0.7)	124 (1.1)	23 (0.9)	33 (1.3)

## Discussion

To our knowledge, this is the first study to describe real-world care patterns of patients with non-IPF chronic fibrosing ILD with a progressive phenotype. Our findings illustrated that patients with a progressive phenotype have high multimorbidity burden and high prevalence of other diseases (e.g. diabetes, COPD, OSA). On one hand, these comorbid conditions may affect the progression of fibrosis ILD. For example, diabetes may influence the progression or the onset of a progression through hyperglycemia-associated pulmonary inflammation [15, 16]. On the other hand, a progressive phenotype may increase the risk of comorbidities the same as in patients with IPF. Previous studies among patients with IPF suggested that these patients are at higher risk of heart failure [17], pulmonary hypertension [17, 18], and OSA [19, 20].

Another important finding of this study is that almost 60% of patients with a chronic fibrosing ILD with a progressive phenotype had a diagnosis of COPD. Moreover, almost half of the included patients in this study had a history of tobacco use. Hence, it is not surprising that patients with a progressive phenotype may also have COPD. It is not certain whether pulmonary fibrosis and COPD are two different diseases associated with tobacco use or if instead they represent a distinctive phenotype of a subset of patients referred to as “combined pulmonary fibrosis emphysema” [21, 22]. Another possibility behind high prevalence of COPD among patients with a progressive phenotype may be related to the misdiagnosis of a progressive phenotype and COPD due to common symptoms (e.g. cough, dyspnoea) [23]. For differentiation between these conditions, the involvement of a pulmonologist in the diagnosis process is crucial.

Two out of three patients with a progressive phenotype had at least one pulmonology visit during baseline. Moreover, this study illustrated that about 71% of patients had at least one pulmonologist visit during follow-up. Pulmonologists play an integral role in diagnosis and management of disease [8]. Expert interviews suggest that pulmonologists are responsible for the diagnosis of this condition [10, 24]. Similarly, a real-world data analysis study in the United States found that almost 75% of patients with ILD visit a pulmonologist at least once a year [10]. For the subset of patients with autoimmune conditions, about 40% patients had both pulmonologist and rheumatologist visits. Currently, the gold standard for the diagnosis of an autoimmune ILD includes the involvement of both pulmonologists and rheumatologists [25, 26]. Patients with mild ILD can be managed solely by rheumatologists, while patients with a progressive phenotype need involvement of pulmonologists [27].

Another finding of this study is that less than half of included patients had HRCT and pulmonary function tests during both baseline and follow-up. These proportions may indicate that both pulmonary function tests and HRCT are underutilized among the patients with a progressive phenotype in the United States. For diagnosis and monitoring of patients with ILDs, using both HRCT and pulmonary function tests are necessary [28]. In a physician survey conducted in United States, Europe, and Japan, common follow-up tests, including pulmonary function tests and HRCT, are suggested every 6 months [10] The main role of HRCT is to identify radiographic patterns that such as usual interstitial pneumonia (UIP), organizing pneumonia (OP) and nonspecific interstitial pneumonia (NSIP) [8]. Following lung function testing and clinical workup, identification of radiologic pattern is sufficient to clinically diagnose most types of ILD (e.g., autoimmune-ILD, HP, etc.) even without a lung biopsy [8, 29].

Adrenal cortical steroids, specifically oral corticosteroids, was the most used medication class during the baseline and follow-up period in this study. This class of medications decreases inflammation but can lead to harmful long-term side effects [30]. In the past, oral corticosteroids were the choice medication for treating systemic sclerosis ILD (SSc-ILD), a type of autoimmune ILDs [31]. Although oral corticosteroids have been used for autoimmune ILDs based on clinical experience, there are no controlled clinical trials of these drugs. Because of concerns about the increased risk of scleroderma renal crisis related to the use of high-dose corticosteroids, only low-dose therapy (20 mg daily) with oral corticosteroids are recommended for patients with SSc-ILD [32]. For other types of ILDs with a progressive phenotype, data are even more limited and variable.

Another finding of this study was that patients with a progressive phenotype had higher percentage of using non-biological DMARDs, immunomodulators, and biological DMARDs during follow-up compared to baseline. Furthermore, the percentages of use of mycophenolate mofetil was almost doubled during follow-up compared to baseline for all patients and those with autoimmune condition. For autoimmune ILDs mycophenolate has emerged as the standard treatment [33]. The use of mycophenolate in autoimmune-ILD has been investigated in a number of case reports, retrospective studies, and prospective RCTs [34, 35]. Moreover, this study illustrated that the percentages of methotrexate and anti TNF-alpha agents (e.g. infliximab, adalimumab) use were decreased during follow-up compared to baseline. This reduction of use may be related to the concerns about potential pulmonary toxicity related to the use of anti TNF-alpha and methotrexate among patients with ILD conditions [36].

### Limitations

First, the data source did not have any clinical information on symptoms, pulmonary function tests results and imaging findings. Therefore, proxies for progression were used for identification of the progression phenotype based on the literature. Second the last year of available data was 2019. Therefore, this study did not identify the use of nintedanib, the only FDA approved medication for chronic fibrosing ILDs with a progressive phenotype [12]. Third, this study did not capture the severity of diseases, type of lung biopsy, and type of pulmonary function test, due to the nature of administrative claims database. Lastly, this study only included US commercial and Medicare health plan enrollees; therefore, the findings are most applicable to insured US patients and may not be generalizable to other populations.

## Conclusions

This study is the first to characterize care patterns of patients with chronic fibrosing ILD with a progressive phenotype. The disease complexity of chronic fibrosing ILD with a progressive phenotype creates significant challenges for clinicians. Moreover, the rarity of this condition limits efforts to conduct more real-world studies to understand care patterns of patients with a progressive phenotype. Comprehensive testing is essential for diagnosis of this condition and diagnostic tests are underutilized for these patients. Accurate diagnosis and treatment require involvement of pulmonologist for all patients with a progressive phenotype which needs attention. For subset of patients with autoimmune conditions a multidisciplinary approach, incorporating rheumatologists and pulmonologists is necessary. Treatment for chronic fibrosing ILDs with a progressive phenotype consists mostly of anti-inflammatory and immunosuppressive agents, which is expected to change following the approval of nintedanib with the indication for chronic fibrosing ILD with a progressive phenotype.

## Supplementary Information

**Additional file 1: Appendix.** Codes list.

## Data Availability

The proprietary data that used in this study are available from Optum Research Database (ORD) but restrictions apply to the availability of these data, and so they are not publicly available. Please contact Optum for information on licensing the data.

## Abbreviations

COPD: Chronic obstructive pulmonary disease
DLco: Diffusing capacity of the lungs for carbon monoxide
FVC: Forced vital capacity
HP: Hypersensitivity pneumonitis
HRCT: High-resolution computed tomography
ICD-10-CM: International Classification of Diseases, 10th Revision, Clinical Modification
ILD: Interstitial lung disease
IPF: Idiopathic pulmonary fibrosis
SD: Standard deviation
SSc-ILD: Systemic sclerosis-associated interstitial lung disease
UIP: Usual interstitial pneumonia
CPT-4: Current Procedural Terminology, 4th Edition
ICD-10-PCS: International Classification of Diseases, 10th Revision, Procedure Coding System
NDC: National Drug Code
